# Low-dose rituximab is no less effective for nephrotic syndrome measured by 12-month outcome

**DOI:** 10.1007/s00467-018-4172-3

**Published:** 2018-12-18

**Authors:** Andrew P. Maxted, Rebecca A. Dalrymple, Denise Chisholm, John McColl, Yincent Tse, Martin T. Christian, Ben C. Reynolds

**Affiliations:** 1 Nottingham Children’s Hospital Renal and Urology Unit, Nottingham, UK; 2 Department of Paediatric Nephrology, Royal Hospital for Children, 1345 Govan Road, Glasgow, G51 4TF UK; 3 Great North Children’s Hospital, Newcastle Upon Tyne, UK; 4 School of Mathematics and Statistics, University of Glasgow, Glasgow, UK

**Keywords:** Rituximab, Nephrotic syndrome, Dosing

## Abstract

**Objective:**

Rituximab is an effective treatment for children with steroid dependent or frequently relapsing nephrotic syndrome. The optimum dosing schedule for rituximab has not been established. We hypothesized that a single low dose of 375 mg/m^2^ would have comparable outcomes to higher doses in reducing the frequency of relapse and time to B cell reconstitution.

**Methods:**

We conducted a multicenter retrospective observational cohort study of children with steroid-sensitive frequently relapsing nephrotic syndrome. Data were extracted from clinical records including the dates of diagnosis, treatment, relapses, the use of concomitant immunosuppression, and lymphocyte subset profiling. Patients treated earlier received variable doses of rituximab, although typically two doses of 750 mg/m^2^. Later, patients received the current regimen of a single dose of 375 mg/m^2^. The primary outcome was an absence of clinically confirmed relapse 12 months following rituximab administration. Secondary outcomes were median time to relapse, probability of being relapse-free at 6 and 24 months and time to reconstitution of CD19^+^ B cells.

**Results:**

Sixty patients received 143 courses of rituximab. Seven different dosing regimen strategies were used, ranging between 375 and 750 mg/m^2^ per dose, with administration of 1–4 doses. There was no significant difference in event-free survival at 12 months between dosing strategies. The median time to reconstitution of B cells was not significantly different between groups.

**Conclusions:**

Use of a single low-dose regimen of rituximab in the management of frequently relapsing nephrotic syndrome does not affect the probability of relapse at 12 months or time to B cell reconstitution compared to a conventional higher dose.

**Electronic supplementary material:**

The online version of this article (10.1007/s00467-018-4172-3) contains supplementary material, which is available to authorized users.

## Introduction

Idiopathic nephrotic syndrome (NS), defined as heavy proteinuria (protein: creatinine ratio greater than 200 mg/mmol) with hypoalbuminemia and clinically detectable edema, is the commonest glomerular disease in childhood, affecting up to 16 per 100,000 children [1]. Histologically, most children have minimal change disease (MCD); initial sensitivity to corticosteroids is used as a prognostic tool [2, 3].

The exact pathogenesis of nephrotic syndrome is unclear, with a combination of genetic predisposition, circulating factors, environmental/infective triggers and other mechanisms hypothesized [4–6]. Eighty to 90% of patients respond to oral corticosteroid therapy, entering remission within 4 weeks. Up to 50% of patients will have frequently relapsing disease (FRNS)—having two or more relapses within a 6-month period—and/or steroid dependent disease (SDNS)—relapses on corticosteroids or within 2 weeks of withdrawal [7, 8].

Additional immunosuppressive agents are often required to minimize steroid toxicity in FRNS/SDNS patients. Examples include the anti-helminthic levamisole, alkylating agents such as cyclophosphamide, calcineurin inhibitors (CNIs) such as ciclosporin or tacrolimus and anti-proliferative agents like mycophenolate mofetil (MMF). None are free of adverse effects or the need for regular monitoring, and all place a concordance burden on the patient and family. Some patients continue to frequently relapse and require corticosteroids despite multiple adjunctive therapies; managing these patients is often a challenge.

Rituximab (RTX) is a chimeric murine-human–anti-CD20 monoclonal antibody, which inhibits CD20-mediated B cell proliferation and differentiation. Developed initially for treatment of B cell non-Hodgkin’s lymphoma [9], RTX has found utility in other diseases including connective tissue disorders and nephrotic syndrome [10, 11]. Usage in FRNS and SDNS has been proven to induce remission and reduce steroid load [12–14].

The optimum dose of RTX in NS has not been determined, with initial dosing strategies based on an established regimen used in lymphoma management. In the UK, a national policy statement recommends two doses of 750 mg/m^2^ on day 1 and 15 [15]. More recently a single dose of 375 mg/m^2^ has been shown to be effective, with even lower doses used in a more recent study [16–18]. The indications for repeated administration of RTX and the timing of such in relation to avoidance of relapse is also unclear.

Based on our collective experience of RTX usage, we hypothesized that a single dose of 375 mg/m^2^ had a similar rate of relapse compared to the standard recommended treatment of two doses of 750 mg/m^2^.

## Patients and methods

This was a multicenter, retrospective, observational, cohort study. Children with NS in Scotland, the North-East of England and the East Midlands, East of England, and South Yorkshire Network were identified via electronic databases and clinician recollection. All children with SDNS or FRNS who received at least one dose of RTX from January 1, 2007, to January 1, 2017, were included. Patients with initial steroid resistance or a subsequent diagnosis other than MCD or focal segmental glomerulosclerosis (FSGS) on biopsy were excluded. Administration of other immunosuppressive therapies was not an exclusion criterion. Additional immunosuppressive therapies were used according to local protocols (adapted from KDIGO guidance) [19].

Clinical and electronic records were reviewed to collect data including basic demographics, date of initial diagnosis, histological diagnosis (if available), date of first administration of RTX, age at first administration of RTX, date of subsequent courses, date of clinical relapse, use of concomitant immunosuppression and results of all measurements of CD19^+^ B cells from lymphocyte subset profiling.

RTX (MabThera™) was administered as per local protocols (supplementary information Table 1). A course of RTX was defined as between one and four doses given within a 4-week period. High dose was defined as 1.5 g/m^2^ (administered as 750 mg/m^2^ in two doses or 375 mg/m^2^ in four doses). Low dose was defined as 375 mg/m^2^ given once. Intermediate doses were all other regimens.

The primary outcome was the probability of remaining relapse-free 12 months following RTX administration. Relapse was defined on standard clinical criteria (supplementary Table 2). Secondary outcomes included the time from last dose of RTX to B cell reconstitution (defined as CD19^+^ B cells greater than 0.2 × 10^9^/L), the 6 and 24-month probabilities of relapse, and the requirement for additional immunosuppression following RTX administration.

Patients were followed up for a maximum of 24 months and data censored beyond this time point.

### Statistical analysis

Event-free survival (EFS) to the fixed end points of 6, 12, and 24 months was analyzed using chi-squared tests of association between treatment group and outcome. EFS up to 24 months was analyzed using Kaplan-Meier survival curves, constructed using either the time to relapse (if less than 24 months) or a censored time at last known, event-free follow up or at 24 months (whichever was sooner). Children who received a prophylactic dose of RTX prior to relapse were treated as censored at the date of administration of the prophylactic dose. Log rank tests were carried out on the same basis. Analysis was carried out to compare the dosing strategies, first versus subsequent course of treatment, number of concomitant immunosuppressants, age at diagnosis (either “typical”, 2–11 years, or “atypical,” < 2 years and > 11 years) and sex of patient. As many patients received more than a single course, all data were also analyzed for the first course only, to limit usage of non-independent data and exclude carryover effects.

CD19^+^ B cell depletion time was not defined as an a priori outcome during our study. CD19^+^ B cell values were measured at variable intervals in different patients so the exact time that they reached > 0.2 × 10^9^/L was not known. Therefore, data were analyzed using three definitions for time to reconstitution: earliest possible time (1 day after the last measurement < 0.2 × 10^9^/L); latest possible time (1 day prior to the measurement of > 0.2 × 10^9^/L); the mean time between these two. Analysis of B cell reconstitution was by Kaplan Meier survival curves and log rank tests. A further analysis was performed using time until CD19^+^ B cell count returned to > 1%; however, this was less informative since the required data were only available for the patients from two centers.

Statistical analyses were performed using Minitab version 17 and R 3.4.0.

## Results

A total of 60 patients with a diagnosis of FRNS/SDNS received at least one dose of RTX between January 1, 2007, and January 1, 2017. Thirty-eight (63.3%) patients were male. Fifty-nine patients received their first course of RTX in our centers, with one patient transferring into the region having previously received one course elsewhere. There were a total of 143 courses of RTX. Median age at diagnosis was 4 years (range 1–14 years) and the median age of administration of the first dose of RTX was 11 years (range 4–17 years). Forty-one patients had a histological diagnosis; 36 showed minimal change disease, with the remaining five showing FSGS. Table 1 summarizes patient characteristics and dosing strategies.

**Table 1:** Patient characteristics and dosing strategies

Patient characteristics		
Total number of patients		60
Male		38
Median age at onset of nephrotic syndrome (years) (range)		4 (1–14)
Median age at first dose of RTX (years) (range)		11 (4–17)
Median time from diagnosis to 1st dose (years)		6
Biopsy result		
Minimal change disease (MCD)		36
No biopsy performed		19
FSGS		5
Total number of RTX courses		
One		19
Two		17
Three		11
Four		5
Five		8
Dosing strategies (all doses)		
750 mg/m^2^ × 2	High dose	13
375 mg/m^2^ × 4		2
750 mg/m^2^ × 1	Intermediate dose	11
500 mg/m^2^ × 2		3
500 mg/m^2^ × 1		7
375 mg/m^2^ × 2		4
375 mg/m^2^ × 1	Low dose	103
Dosing strategies (first dose)		
750 mg/m^2^ × 2	High dose	12
375 mg/m^2^ × 4		2
750 mg/m^2^ × 1	Intermediate dose	1
500 mg/m^2^ × 2		3
500 mg/m^2^ × 1		0
375 mg/m^2^ × 2		1
375 mg/m^2^ × 1	Low dose	40

*RTX* rituximab

### Time to relapse

#### Whole cohort (including subsequent and prophylactic doses)

**Fig. 1:**
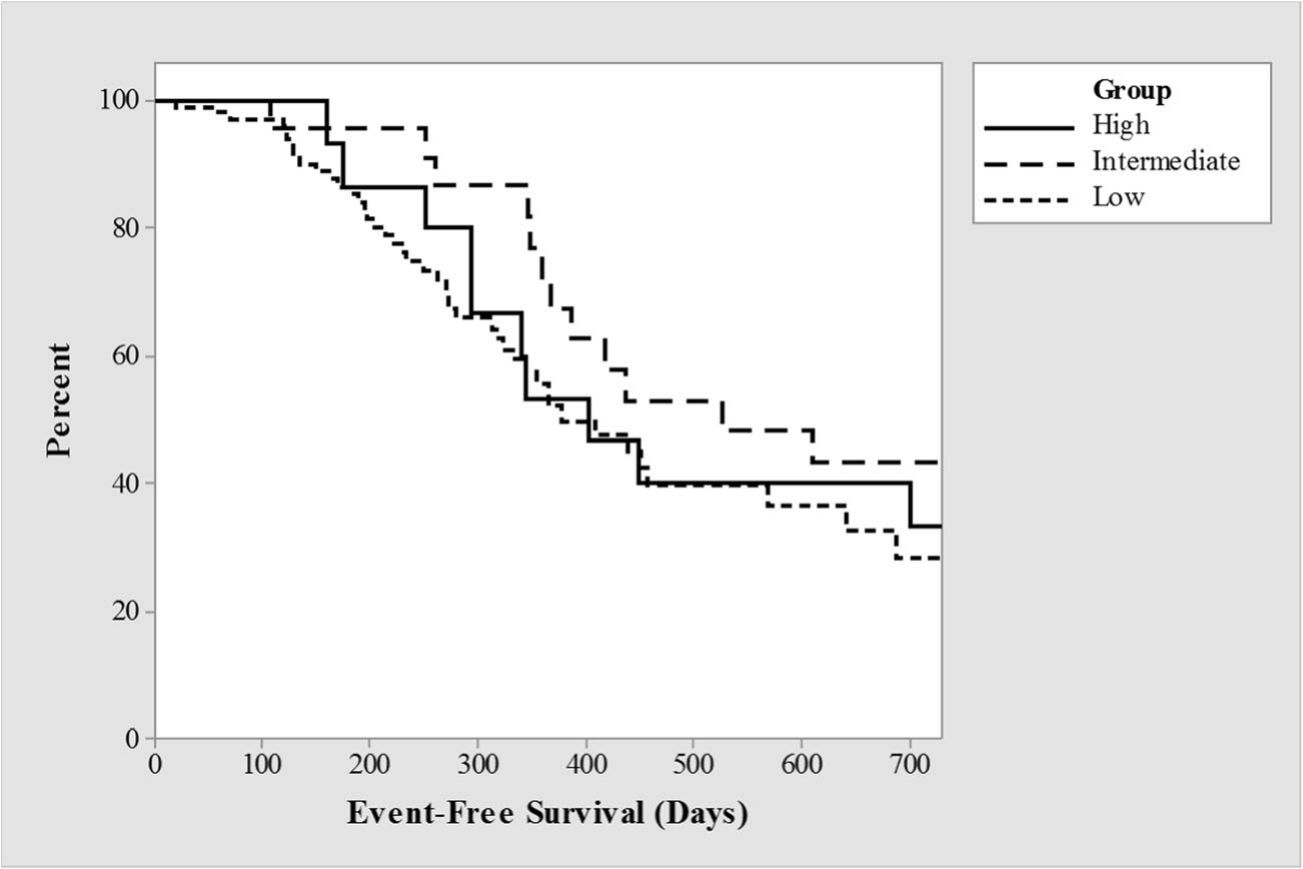
Kaplan-Meier estimates of relapse-free survival curves by dosing schedule, *p* = 0.32

There was no significant difference in the incidence of clinical relapse at 12 months between the different dosing groups (*p* = 0.15) with an event-free survival of 53%, 71%, and 47% for the high, intermediate, and low-dose groups respectively. Survival analysis did not demonstrate significance on cumulative event-free survival by dosing regimen (*p* = 0.32) (Fig. 1).

During the study 26 patients received a subsequent dose of RTX prior to a clinical diagnosis of relapse, i.e., prophylactically, at a median of 179 days (range 51–540 days) after their previous dose. These patients were censored at the time of their prophylactic dose for the purpose of EFS. Dosing for (non-prophylactic) subsequent doses was not the same across the cohort, with variable time-frames, dosing and correlation with B cell numbers. We therefore analyzed patients after the first dose only to eliminate confounding variables.

#### First dose of RTX only

Following their initial course of RTX, 40 (68%) of 59 patients had at least one relapse. Nine (15%) patients had 24-month follow-up with no relapse. Five (8%) patients had no relapse, but with follow-up less than 24-months, with data censored at latest follow-up. Five (8%) patients received a prophylactic second dose prior to clinical relapse, with data censorship at the time of administration of the second course, Table 2.

**Table 2:** 6, 12, and 24-month event-free survival (EFS) at different doses—first treatment only

Dosing strategy	Total number of doses	6-month EFS	12-month EFS	24-month EFS
High	14	12/14 (85.7%)	7/14 (50.0%)	4/14 (28.6%)
Intermediate	5	5/5 (100%)	4/4 (100%)	3/4 (75.0%)
Low	40	30/37 (81.1%)	13/34 (38.2%)	2/30 (6.7%)
Chi-squared value		1.213	5.608	12.069
*p* value		NS	0.06	0.0024

It is important to note that the intermediate group contains only 5 patients. This limits statistical comparisons and as such further chi-squared analysis was performed by grouping the intermediate group with the higher dose, this produces similar results at 6 months (chi-squared *p* = NS), 12-months (*p* = 0.12) and 24 months (0.0056).

**Fig. 2:**
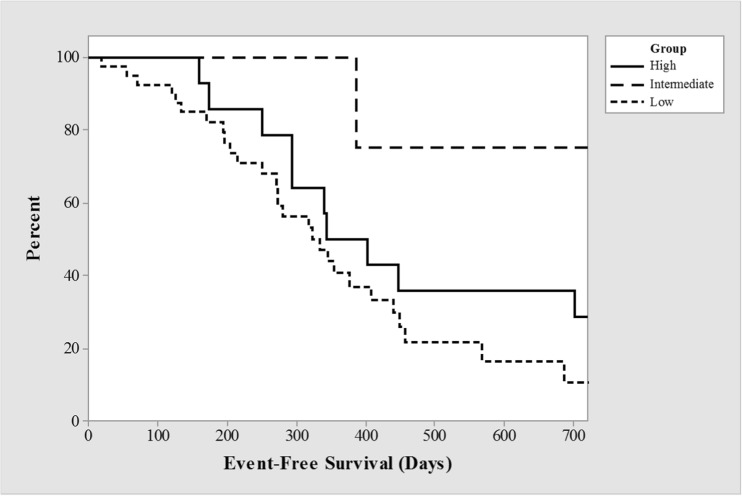
Kaplan-Meier survival curves of time relapse-free survival by dose schedule for first dose of rituximab (RTX) only (*p* = 0.05)

The incidence of relapse in the 24 months following the first administration of RTX was different between dosing strategies; high (71%), intermediate (25%), and low (72%) groups (supplemental Table 3). However, there were only 5 patients whose first course of treatment was at the intermediate dosing level and a single-patient relapsed. The median time to relapse after the first dose by Kaplan-Meier survival analysis was 344, > 720 and 334 days for high, intermediate, and low-dose groups. A log-rank test comparing the survivor curves approached significance (*p* = 0.05), though the smaller number of patients for comparison of initial dose limits the conclusions that may be drawn from this group (Fig. 2).

**Fig. 3:**
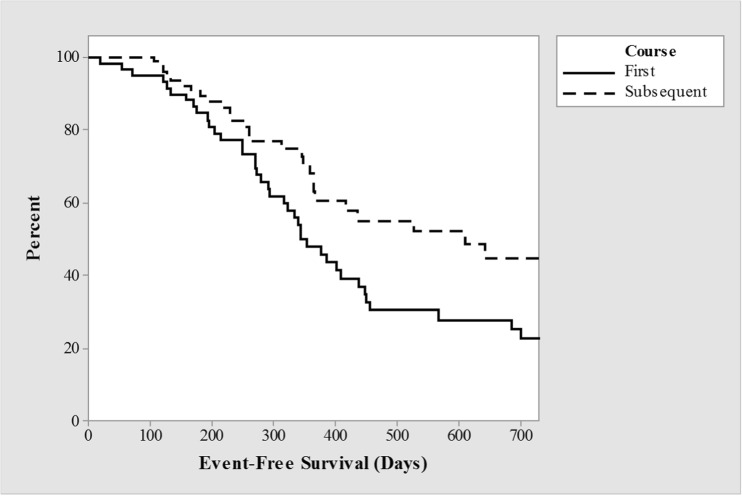
Kaplan-Meier estimates of survivor curves by course of treatment, *p* = 0.02

Adjustment for single variables using Cox proportional hazard models only demonstrated significance of dosing schedule for EFS when considering first-dose data alone (*p* = 0.02). Other variables showed no significant difference, including treatment center (*p* = 0.97), sex (*p* = 0.90) and age (*p* = 0.98). When all dose administrations were modeled, the difference between dosing regimens became non-significant (*p* = 0.29). EFS between the first dose and subsequent doses is significant (*p* = 0.02), suggesting a cumulative effect, Fig. 3.

### B cell reconstitution

Excluding the 26 courses given prior to a prophylactic dose, only 56 of 117 (48%) courses demonstrated reconstitution. Seven of 117 (12%) did not have any subsequent assessment of B cell populations. In 54 courses, measurements of B cell populations did not demonstrate reconstitution at last follow-up. Three courses had CD19^+^B cell numbers >0.2 × 10^9^/L within 1 month of administration (measured at days three, seven, and 23); then, subsequent lymphocyte profiling without re-dosing demonstrated depletion. In two of these three courses, B cells did not demonstrate reconstitution during follow-up.

**Fig. 4:**
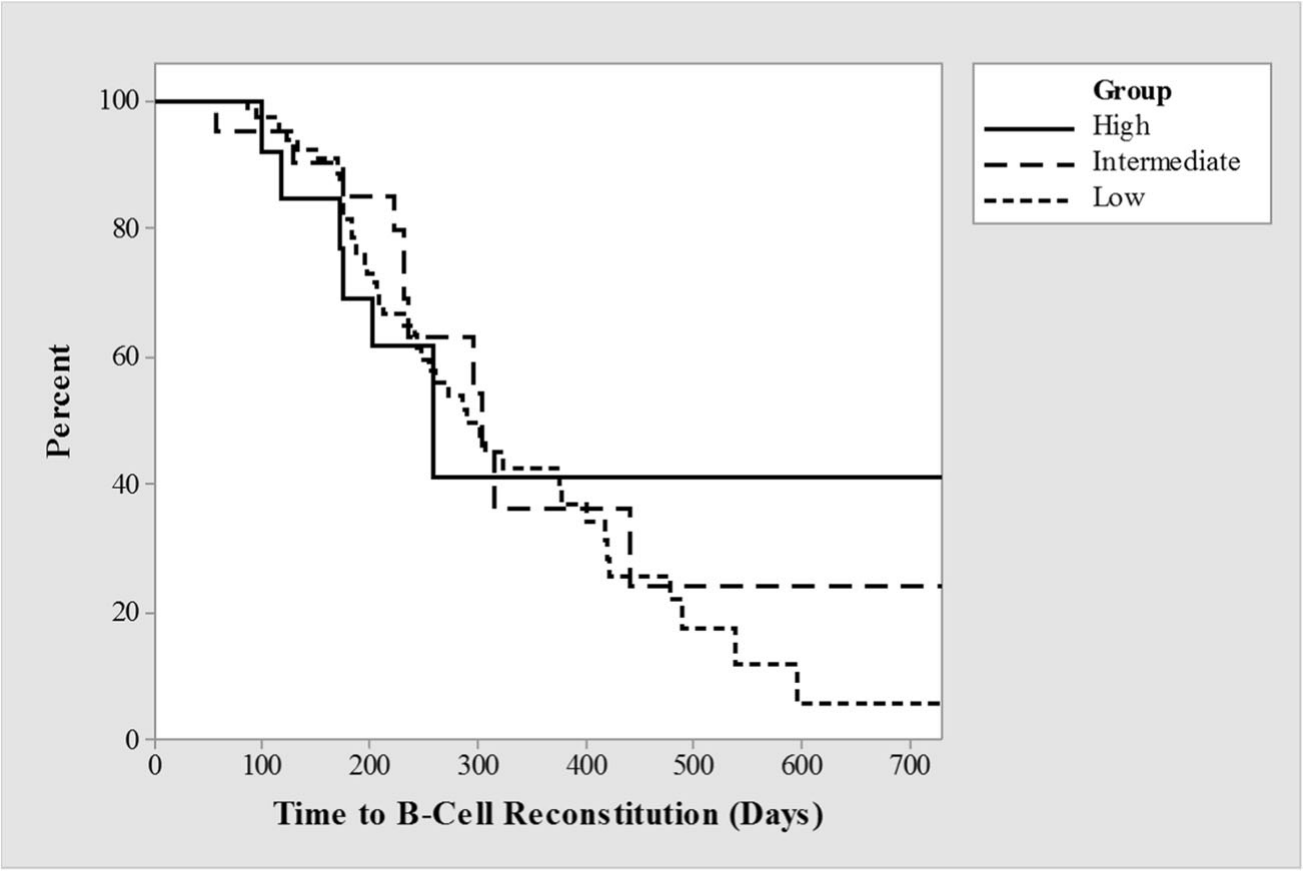
Kaplan-Meier survival (by interval censored analysis) of the time to B cell reconstitution, > 0.2 × 10^9^/L between high, intermediate, and low groups

For the overall cohort, the median time to reconstitution of CD19^+^ B cells (> 0.2 × 10^9^/L) by survival curve analysis was 295 days. There was no significant difference between the high, intermediate and low-dose schedules (259, 304 and 290 respectively) (Table 3, Fig. 4). Excluding courses never demonstrating reconstitution, the median time was 226 days.

**Table 3:** Time to B cell reconstitution (days)

Dosage	Number of doses with data available	Median time to B cell reconstitution (days) > 0.2 × 10^9^	Number of doses with data available	Median time to B cell reconstitution (days) > 1%
High dose	14	259	12	342
Intermediate dose	25	304	12	314
Low dose	96	290	37	217

There was no difference in reconstitution times between the different dosing groups irrespective of how time to reconstitution was defined; (i) time to last negative result (log rank test, *p* = 0.93); (ii) time to first positive result (*p* = 0.89); (iii) time to mid-point between these two tests (*p* = 0.98).

Some groups defined B cell reconstitution as a subset population percentage rather than an absolute number, i.e., CD19^+^ B cells > 1% of the total. Re-analysis using this alternative definition did not alter non-significance; the median time to reconstitution was 342, 314, and 217 for the high, intermediate and low-dose groups. These data were only available for patients from two centers and as such conferred smaller patient numbers.

### Safety

Minimal severe adverse events were observed in this patient cohort. Documentation of minor infusion reactions was occasionally noted, but in only a single case was it clinically significant, requiring discontinuation of the infusion. The same patient subsequently had an anaphylactic reaction to a second dose; further dosing was not attempted. Two patients developed persistent asymptomatic hypogammaglobinemia, one receiving replacement intravenous immunoglobulin, one being managed expectantly. There were no recorded episodes of sepsis.

### Concomitant immunosuppression

Though a priori outcomes included concomitant immunosuppression, multiple patients were missing information. Data were available from two out of the three centers. As we have shown above, single variable analysis using Cox proportional hazard models showed no significant difference between treatment centers (*p* = 0.97) when analyzing EFS. Analysis of these patients (39 at time of first RTX) showed all to be on prednisolone. Choice of concomitant immunosuppression varied but included a combination of CNIs and MMF. There was no statistical significance in the number of different immunosuppressants at the time of first dose between dosing regimens (*p* = 0.060). Multi-variable analysis using Cox proportional hazard models was used to test for any effect of concomitant immunosuppression in addition to dosing regimen on EFS. The number of concomitant immunosuppressants at both time of first dose of RTX (*p* = 0.42) and at 6-month post-dose (*p* = 0.24) did not demonstrate significance.

## Discussion

This multicenter study demonstrates that a single dose of RTX of 375 mg/m^2^ has a similar outcome rate in suppressing CD19^+^ B cells and maintaining remission in pediatric patients with SDNS or FRNS compared to higher doses (up to 1.5 g/m^2^) at 6 months, but this is less convincingly the case at 12 months. At 24 months, low dose is inferior to high and intermediate dosing. Despite very low numbers in the intermediate group, statistically significant improvement in relapse-free survival was seen. This study is underpowered to definitively determine whether this is related to the low patient numbers within that group but highlights that low-dose regimens may not be as effective in the longer term.

Rituximab usage in pediatric nephrotic syndrome was first reported in 2004 [20], using 375 mg/m^2^ weekly for 4 weeks. Subsequently, RTX has been widely reported to be efficacious in FRNS/SDNS with multiple dosing strategies [12, 14, 18, 21–25]. The largest randomized placebo-controlled trial of RTX in NS administered four weekly doses of 375 mg/m^2^, reporting a median relapse-free period of 267 days in the RTX group compared to 101 days with placebo (hazard ratio 0.27, *p* = < 0.0001) [14]. Two meta-analyses of RTX in pediatric NS both demonstrated an improved relapse-free survival with RTX compared to other immunotherapies (relapse-free survival hazard ratio of 0.49 (*p* = 0.03)) [26] and relative risk of remaining relapse-free at 6 months of 5.25, *p* < 0.0001) [27].

Fujinaga reported 24-month relapse rates on a low-dose regimen, reporting 91% relapse (39 patients) at a median of 586 days [28]. Greater risk of relapse was associated with younger age of administration and more rapid reconstitution of B cells [28].

In our cohort, the statistical significance of increased relapse rate at 24 months for low dose compared to intermediate dose is highly influenced by the small numbers in the latter group—only 1/5 patients relapsed (Fig. 2). If a second patient had relapsed, significance would have been negated. Therefore, this prevents any firm conclusion that the low-dose regimen is non-inferior at 24 months.

Hogan et al in their study compared 100 mg/m^2^ with 375 mg/m^2^ and 750 mg/m^2^ (as two divided doses). They showed a slightly shorter time to B cell reconstitution with the 375 mg/m^2^ group compared with our cohort (150 days vs 290 days), but a longer event-free survival at 12 months (59% vs 38%). They concluded increased dosing is associated with increased time to B cell reconstitution, which is in contrast to our results [18]. It should be noted that they also had small numbers in their low-dose (8) and high-dose groups (18).

Weaning/cessation of other immunosuppression is often performed following RTX administration, though the evidence supporting this approach remains limited. Some centers are reported to have discontinued all immunosuppression while the B cell number is suppressed [12]. Though we intended to investigate this relationship in detail, we only had data available from two centers. This demonstrated no significant effect on EFS when immunosuppression at 6 months was added to treatment groups in a Cox proportional hazards model. There was a trend towards longer EFS in patients on less concomitant immunosuppression (both prednisolone and other agents). We suggest this reflects patients with a better clinical course following RTX being more likely to have reduction in immunosuppression. However, due to the small numbers where data is available, firm conclusions cannot be drawn.

The definition of CD19^+^ B cell reconstitution is not standardized; some authorities report a percentage of the B cell population, others use an absolute number. We have presented our data in both formats (where available). The median time to reconstitution is comparable to other studies (Table 4) [12, 14, 16–18, 21, 24, 29].

**Table 4:** Comparative relapse and remission data with other reports following first dose of rituximab (RTX)

Study	Year	Diagnosis	Dose	No. patients	Duration of CD19^+^ B cell depletion	6 month EFS	1 yr. EFS	Median time to relapse*
Maxted et al	2017	FRNS or SDNS	Variable: 375 mg/m^2^ to 1.5 g/m^2^	59	Median depletion 10 months	84%	46%	11 months
			1 × 375 mg/m^2^	40	Median depletion 10 months	81%	38%	11 months
Niu et al	2016	SDNS	Single dose 375 mg/m^2^	19	Mean depletion time 3 months	74%	63%	5 months
Ravani et al	2015	SDNS	Single dose 375 mg/m^2^	15	Mean depletion 6 months	93%	66%	18 months (between RTX treatments)
Iijima et al	2014	FRNS or SDNS	4 doses 375 mg/m^2^	20	Median depletion lasted 5 months	–	15%	9 months
Tellier et al	2013	SDNS	1–4 doses 375 mg/m^2^ (10 patients received 4)	18	–	–	–	13 months
Sellier-Leclerc et al	2012	SDNS	1–4 doses 375 mg/m^2^ (15 patients received 4)	30	Median depletion 8 months	–	–	–
Ravani et al	2011	SDNS	1–2 doses of 375 mg/m^2^	27	–	50%	25%	–
Sinha et al	2015	SDNS	1–4 doses of 375 mg/m^2^	101	–	92%	58%	16 months
Hogan et al	2018	SDNS	1 dose of 375 mg/m^2^	35	Median depletion 5 months	–	59%	–

*EFS* event-free survival, *SDNS* steroid-dependent nephrotic syndrome, *FRNS* frequently relapsing nephrotic syndrome

*Excludes patients receiving prophylactic dosing and those with no subsequent relapse

Following the initial dose of RTX, 19 patients (32%) of our cohort did not relapse. This occurred across all dosing groups. Current practice in the UK is often to consider RTX after failure of other immunosuppressive strategies; this demonstrates the potential efficacy of low-dose RTX in “complicated” nephrotic syndrome. If a single intravenous dose leads to long-term remission in a quarter of patients, this could avoid the long-term accrual of risk currently associated with other agents used and has utility in patients with concordance issues. Arguably, it might be better used earlier in FRNS/SDNS algorithms [30].

Prophylactic use of RTX has been described in a time-dependent fashion [31]. Though reducing the likelihood of relapse, prophylactic dosing leads to administration to patients never destined to relapse. Further work is required to determine which patients may benefit from prophylactic administration. We agree with a recent review article, suggesting large-scale trials to ascertain the correct dosing regimen to balance effectiveness with safety and cost-benefits [32].

Rituximab is not a benign medication. Long-term adverse effects remain unknown due to the relative novelty of the drug. Minimization of drug exposure may reduce overall adverse effects, though these data are lacking. RTX is a relatively expensive medication; usage of the low-dose regimen reduces immediate medication costs by 75%, before accounting for savings from reduced hospital attendance for repeated infusions.

Limitations of this study include the retrospective and observational nature of the cohort, with no randomization of therapy or blinding. Selection and systematic bias cannot be excluded. Patients treated earlier in the study were more likely to receive the larger doses. Other aspects of their treatment may also have differed, e.g., choice of CNIs. Many of the low-dose group had a shorter follow-up so required censoring for last follow-up and relapse-free time. Withdrawal of other immunosuppression was variable and managed by individual clinicians. Data were not collected on minor adverse events for all patients, so it is not possible to comment on whether the lower dose regimen had fewer self-limiting adverse effects. Finally, due to a number of patients receiving a combination of high, intermediate and low doses, demographic bias between dosing schedules cannot be taken into consideration.

Strengths of this study are the inclusion of multiple patients with a relatively homogenous population and a small number of clinicians. Sixty pediatric patients receiving 143 courses of RTX represents one of the largest cohorts reported to date.

## Conclusion

A low-dose regimen for RTX of a single dose of 375 mg/m^2^ is similarly effective to higher dosing regimens in the treatment of steroid sensitive, frequently relapsing nephrotic syndrome at 6 and 12 months. However, this is not the case at 24 months and as such a higher dose may be beneficial for the initial treatment when longer follow-up is complete, but this study was underpowered to confirm conclusively. As many centers are reporting outcomes with a lower dose regimen, further work is required to establish the minimum effective dose, the role of prophylactic dosing, and the role of concomitant immunosuppression.

## Electronic supplementary material

ESM 1 (DOCX 19 kb)
