# Supplementary material for: Low-dose rituximab is no less effective for nephrotic syndrome measured by 12-month outcome
Source: Pediatr Nephrol. 2018 Dec 18;34(5):855–63. doi: 10.1007/s00467-018-4172-3 (PMC6424916; doi:10.1007/s00467-018-4172-3)
Supplement: Supplementary file 1 — (DOCX 19 kb) [file 467_2018_4172_MOESM1_ESM.docx]

**Supplementary table 1:** Protocol for Rituximab infusion

| Patients are admitted as a day case, except in situations where travel is so long as to make this inconvenient. No test dose is trialed before administration.  Baseline investigations are done at the time of intravenous access including urea, creatinine, immunoglobulins and CD19^+^ counts.  Patients are pre-medicated with oral Paracetamol (15mg/kg max 1g), Intravenous (IV) Chlorphenamine (1-6yr 2.5mg, 6-12yr 5mg and >12yr 10mg), IV Methylprednisolone (60mg/ m^2^) and Hydrocortisone (<6months 25mg, 6months-6yr 50mg and >6yr 100mg).  Rituximab (MabThera™) was given at a dose of 375mg/ m^2^ (rounded to nearest 50mg) diluted in 0.9% Sodium Chloride to 1-4mg/ml. The infusion was started at 25mg/hr, and then, if tolerated increased every 30 minutes by 25mg/hr to a maximum rate of 300mg/hr.  Observations; pulse, blood pressure, respiration, temperature and oxygen saturations are monitored every fifteen minutes for the first hour and then hourly after this point if stable and tolerating increased infusion rate.  Patients did not routinely receive antimicrobial prophylaxis for *Pneumocystis jirovecii* |
| --- |

**Supplementary table 2:** Definitions used in study

| FRNS: | Three relapses (including the initial episode) within the first year or two or more relapses in any 6 months thereafter. |
| --- | --- |
| SDNS: | Two consecutive relapses on oral steroids or within two weeks of stopping. |
| Remission: | Three consecutive days of trace proteinuria or no proteinuria |
| Relapse: | Proteinuria of three plus on dipstick for three consecutive days (often occurring, but not exclusively with peripheral edema) |
| Dose of RTX | A single clinical event in which RTX was administered at either 375mg/m^2^ or 750mg/m^2^ |
| Course of RTX | RTX being administered at varying time points, for example 1.5g/m^2^ given as two doses of 750mg/m^2^ two weeks apart |

**Supplementary Table 3:** Relapse frequency by dosing schedule for patients with complete 24 months follow-up

| Dosing schedule | Relapse | Total |
| --- | --- | --- |
| High | 10 | 14 |
| Intermediate | 1 | 5 |
| Low | 29 | 40 |
| All | 40 | 59 |
